# Profiling bitter taste receptors (TAS2R) along the gastrointestinal tract and their influence on enterohormone secretion. Gender- and age-related effects in the colon

**DOI:** 10.3389/fendo.2024.1436580

**Published:** 2024-10-17

**Authors:** Florijan Jalševac, Maria Descamps-Solà, Carme Grau-Bové, Helena Segú, Teresa Auguet, Francesc Xavier Avilés-Jurado, Francesc Balaguer, Rosa Jorba, Raúl Beltrán-Debón, Maria Teresa Blay, Ximena Terra Barbadora, Montserrat Pinent, Anna Ardévol

**Affiliations:** ^1^ Universitat Rovira i Virgili, Department of Biochemistry and Biotechnology, MoBioFood Research Group, Tarragona, Spain; ^2^ Institut d’Investigació Sanitària Pere Virgili (IISPV), Hospital Joan XXIII, GEMMAIR Research Group, Tarragona, Spain; ^3^ Institut d’Investigació Sanitària Pere Virgili (IISPV), Hospital Joan XXIII, Tarragona, Spain; ^4^ Head Neck Tumors Unit, Hospital Clínic de Barcelona, Universitat de Barcelona, IDIBAPS, Barcelona, Spain; ^5^ Department of Gastroenterology, Hospital Clínic de Barcelona, Institut d’Investigacions Biomèdiques August Pi i Sunyer (IDIBAPS), Centro de Investigación Biomédica en Red de Enfermedades Hepáticas y Digestivas (CIBEREHD), University of Barcelona, Barcelona, Spain; ^6^ Institut d’Investigació Sanitària Pere Virgili (IISPV), Hospital Joan XXIII, MoBioFood Research Group, Tarragona, Spain

**Keywords:** bitter taste receptors (TAS2R), human gastrointestinal tract, enterohormones, ageing, colon

## Abstract

Extraoral bitter taste receptors offer intriguing potential for modulating metabolism and the gut-brain axis through dietary interventions. Our understanding of these receptors is limited, and data on their effects on ageing are scarce. The complexity conveyed by their high diversity, low expression levels and species-dependent variability challenges our comprehension. We used real-time PCR to examine the relative abundance of multiple TAS2R across different segments of gastrointestinal mucosa in four human cohorts and related them to enteroendocrine secretions at the colon site. TAS2R14 exhibited the highest expression levels in all analyzed tissues. In contrast, TAS2R39, -38 and -42 consistently exhibited lower expression levels. Ageing was found to upregulate TAS2R4, -5, -13, -20 and GLP-1 mRNA in the descending colon. Stimulating TAS2R14 in Hutu-80 cells induced GLP-1 secretion, while stimulating TAS2R5 modulated GLP-1 and PYY secretion. Given the modifications TAS2R agonists may undergo along the GIT, as well as the distinctive expression patterns and possible functional roles of TAS2R receptors along the intestinal tract, our findings suggest the viability of a targeted strategy aimed at enhancing specific functions to improve health outcomes. This study offers valuable insights into the intricate interplay between bitter taste receptors, gut physiology and potential dietary interventions.

## 1 Introduction

Our organism is profoundly influenced by the composition of our diet, since the foods we consume provide the building blocks for body formation and maintenance and the energy required for numerous physiological processes. Food components also convey information to our bodies about the characteristics and quality of our meals. Though gut-brain communication is well established (1), it is important to also recognize that our gut communicates with our whole body. The gastrointestinal tract (GIT) wall contains numerous proteins that participate in these functions (2). In the past decade, a new group of membrane receptors, known as taste receptors (TASR), has been identified (3). Initially, taste receptors were primarily associated with the tongue, but subsequent research has revealed their presence in several other tissues (4). One location where these extraoral receptors appear is the GIT, where TASR pose as candidates for playing a key role in the detection of food components. The largest group of taste receptors are the bitter taste receptors (TAS2R). Indeed, recent data indicate that there are 26 functional variants of TAS2R in humans [5]. As we can see from the Human Protein Atlas (http://www.proteinatlas.org), the expression levels of extraoral TAS2R tend to be lower than those of other receptors. However, when their stimulation was analyzed by Meyerhoff et al. (5, 6), they found that the effective concentration (EC50) for their ligands is typically in the micromolar range. Considering the movement of food components through the gastrointestinal tract from the mouth to the anus, it is therefore reasonable to consider that the TAS2R present in different cells of the intestinal wall may be stimulated by effective concentrations of ingested compounds or products of food components that have been modified along the GIT.

It is important to mention that the characteristics of bitter taste receptors vary between species. Moreover, if we compare individual species, we find that each has a larger or smaller range of different TAS2R in which, though certain similarities can be found, species-specific characteristics are also present, thus making their story more fascinating (7). The small structural but impactful differences between different TAS2R mean that they exhibit different abilities to bind different compounds, as some show high levels of promiscuity, binding to a variety of compounds, while others show higher selectivity (8). These diverse characteristics make them highly valuable assets for precise taste perception on the tongue. It could therefore be speculated that the presence of diverse TAS2R at other locations may contribute to their specific functions there, though the role of extraoral TAS2R is not yet fully understood (9). Some studies indicate that TAS2R can modulate energy expenditure (EE) (10, 11) and it is suggested that they are involved in the inflammatory intestinal functions (12–14). Furthermore, it has been shown that specific genetic modifications of different taste receptors are important factor in the development of obesity (15). Finally, procedures such as bariatric surgery result in changes in the taste receptors in the lingual taste buds, but also further down the gastrointestinal tract (16).

The gut communicates, among other mechanisms, by secreting diverse range of enterohormones along all its length that allows brain-gut communication. The stimulation of TAS2R has been shown to lead to the enterohormone secretion as summarized by Tuzim et al. (17). Since enterohormones such as GLP-1, PYY, GIP and ghrelin are key regulators of food intake and related metabolic processes, it is reasonable to consider that the TAS2R are key targets for the regulation of whole-body homeostasis.

For a more thorough understanding of the significance of TAS2R receptors throughout the GIT and their potential role in mediating the communication between our diet and our body, it is therefore essential to investigate their distribution along the entire GIT, their specific functions at each location, and the factors that modulate their expression and activity. Knowledge is currently limited regarding how the functionality of these receptors is influenced by potential gender-specific effects, ageing (18), modifications in the context of obesity (19), and other relevant factors. Further studies are therefore needed to assess how these factors influence TAS2R function.

In this study we present data on the relative abundance of several TAS2R at three human mucosal locations. Our aim for this study was to establish a profile of distribution of GIT located TAS2R and to ascertain their role as enterohormone regulators, establishing these receptors as potential targets for interventions. This analysis enables us to examine the different sensitivities to bitter sensing at these sites. More specifically, we focus on the colonic site and investigate the influence of variations in gender, age and location in this particular section of the GIT. We also explore the potential relationship between the presence of TAS2R in the colon and the key enterohormones present in this region.

## 2 Materials and methods

### 2.1 Biopsy sample collections

Included in this study were four distinct groups of patients. The exclusion criteria are summarized in Supplementary data (**Supplementary Appendix A1**). Samples of human cheek mucosa were kindly provided by Joan XXIII University Hospital (Tarragona, Spain). Healthy mouth mucosa samples were collected from male patients (n=25, age=65.2 ± 10.7) diagnosed with head and neck cancer. The tissues were stabilized in RNAlater preservative (Qiagen, Hilden, Germany) and stored at -80°C. This experimental procedure was approved by the Clinical Research Ethics Committee (CEIC) of Joan XXIII University Hospital in Tarragona (PI15/02047).

Jejunum samples were kindly provided by GEMMAIR from IISPV and Joan XXIII University Hospital (Tarragona, Spain). Jejunal biopsy samples were collected from women with obesity (BMI > 40 kg/m2) who had undergone a bariatric Roux-en-Y gastric bypass (RYGB) procedure (n=21, age=46.1 ± 11). The tissues were stabilized in RNAlater preservative (Qiagen, Hilden, Germany) and stored at -80°C. This experimental procedure was approved by the Clinical Research Ethics Committee (CEIC) of Joan XXIII University Hospital in Tarragona (23c/2015).

Mucosal biopsy samples from the ascending and the descending colon were kindly provided by the Hospital Clínic (Barcelona, Spain). Colon biopsy samples were collected from healthy men (n=23) and women (n=25) who had undergone colon cancer screening procedure. The tissues were stabilized in RNAprotect (Cat. No.: 76104, Qiagen, Hilden, Germany) and stored at -80°C. Plasma samples were also obtained. Subjects were divided into two age groups (one younger, age=38.9 ± 6; and one older, age=63.6 ± 6). This experimental procedure was approved by the Drug Research Ethics Committee of Hospital Clínic de Barcelona (HCB/2019/1115).

Another set of colon samples was kindly provided by Joan XXIII University Hospital (Tarragona, Spain). The tissues were collected from patients (12 male and 5 female) with pathologically confirmed colorectal carcinoma (age=63.4 ± 3) who had undergone colon surgery. Non-diseased tissues were excised from both the ascending colon (n=8) and the descending colon (n=9). This experimental procedure was approved by the Clinical Research Ethics Committee (CEIC) of Joan XXIII University Hospital in Tarragona (CEIm 101/2017).

The potential influence of different medications prescribed to the patients has been assessed. No medication was intestine-specific, and just a handful of patients were prescribed proton pump inhibitors for the control of stomach acid (**Supplementary Table S1**). All participants were informed before they provided their written consent to participate in their part of the study.

### 2.2 Colon segment enterohormone basal secretion analysis

After resection, the colon tissues obtained from Joan XXIII University Hospital were transferred to the laboratory within 30 minutes in ice-cold KRB-D-Mannitol buffer (pH 7.4) saturated with 95% O_2_ and 5% CO_2_. The tissues were rinsed, and the serosa and outer muscular layers were removed. After a 10-minute washing period, tissue segments were placed in pre-warmed KRB-D-Glucose bufler 0.1% DMSO with protease inhibitors. The samples were treated for 30 minutes in a humidified incubator (37°C, 5% CO_2_). Media were collected and stored at -80°C.

### 2.3 Total RNA extraction and RT-qPCR

All samples collected from the above patients for gene expression analysis were treated uniformly. RNA extraction was performed using a RNeasy Plus Mini Kit (Cat. No.: 74134, Qiagen, Hilden, Germany) in accordance with the manufacturer’s instructions. Frozen samples were disrupted and homogenized using a TissueLyser LT small bead mill (Qiagen, Hilden, Germany). The quality and purity of the extracted RNA were evaluated using a NanoDrop^®^ ND-1000 spectrophotometer (Fisher Scientific, Madrid, Spain), and then stored at -80°C. cDNA was obtained using a High-Capacity cDNA Reverse Transcription Kit (Cat. No: 4368814, Fisher Scientific, Madrid, Spain).

Quantitative PCR amplification was performed using specific TaqMan probes (list of probes: (**Supplementary Appendix A2**); Applied Biosystems, Waltham, USA) for all TAS2R and for enterohormones quantification (40 ng/μL of cDNA for TAS2R, 4 ng/μL for enterohormones). The relative expression of each gene was compared with that of the control group using the 2–ΔΔCt method and with RPS9 used as reference. Each figure indicates the analysis performed.

### 2.4 Hutu cell studies

#### 2.4.1 Cell culture

Enteroendocrine HuTu-80 (ATCC, HTB-40) cell line was provided by LGCgroup (Barcelona, Spain). The cells were grown in culture flasks (Greiner Bio-One, Frickenhausen, Germany) at 37°C in an atmosphere of 5% CO_2_ and the medium was changed every 2–3 days. The growth medium consisted of EMEM (Cat. No.: 30-2003, ATCC, Manassas, USA) supplemented with 10% v/v heat-inactivated fetal bovine serum (Cat. No.: 12103C, Sigma-Aldrich, Madrid, Spain) and 100 U/mL penicillin-streptomycin mixture (Cat. No.: DE17-602E, Lonza Bioscience, Basel, Switzerland). When confluence reached roughly 80%, the cells were harvested by treatment with 0.25% Trypsin 1 mol/L EDTA (Cat. No.: T3924, Sigma-Aldrich, Madrid, Spain) for 5 minutes, and then split and sub-cultured in fresh medium.

#### 2.4.2 Cellular gene expression analysis

For this experiment, passages 13-17 were used. HuTu-80 cells (200,000 cells/mL) were seeded into individual culture plates (Greiner Bio-One, Frickenhausen, Germany). After 48 hours, cells were washed with cold PBS, then lysed using a buffer provided in the RNeasy Plus Mini Kit (Cat. No.: 74134, Qiagen, Hilden, Germany), and stored at -80°C. The extraction process, RNA to DNA transcription, and gene expression analysis were conducted as described above using the same TaqMan probes and the same reference gene.

#### 2.4.3 Cellular enterohormone secretion analysis

For this experiment, passages 14-17 were used. HuTu-80 cells (200,000 cells/mL per well) were seeded into 24-well plates. After 48 hours, the cells were washed with PBS and treated for two hours with specific treatments: vanillic acid (150 μM) (Cat. No.: V0017, TCI Europe N.V., Zwijndrecht, Belgium), epicatechin (500 μM) (Cat. No.: E1753, Sigma-Aldrich, Madrid, Spain), and peptone (5 mg of protein/mL) (Cat. No.: 70175, Sigma-Aldrich, Madrid, Spain). The stock solutions of the treatments were prepared in DMSO (Cat. No.: SU01581000, Scharlab, S.L., Barcelona, Spain) and then diluted to final working concentrations in glucose-free Krebs–Ringer buffer (KRB) (120 mM NaCl, 5 mM KCl, 2 mM CaCl_2_, 1 mM MgCl_2_, 22 mM NaHCO_3_). The final DMSO concentration was 0.05%. For basal secretion, KRB supplemented with 0.05% DMSO was used. After treatment, the medium was collected and stored at -80°C.

The cells were then lysed with HEPES buffer supplemented with Triton^®^ X-100 (0.1%; Cat. No.: T8787, Sigma-Aldrich, Madrid, Spain) and stored at -80°C. Total protein content by means of a Bicinchoninic Acid (BCA) kit (Pierce, Thermo Fisher Scientific) and Lactate Dehydrogenase Assay (LDH) (QCA, Tarragona, Spain) quantification of cytotoxicity in the cells were both performed, as previously described (20).

### 2.5 Enterohormone quantification

Enterohormone assays were performed using commercial ELISA kits. For both the media obtained from human colon segments and the cellular secretion experiment, GLP-1 (intra-assay variation: 1-2%; inter-assay variation: <12%; Cat No.: EZGLPT1-36k, Millipore, Madrid, Spain) and PYY (intra-assay variation: <10%; inter-assay variation: <15%; Cat No.: FEK-059-02, Phoenix Europe GmbH, Karlsruhe, Germany) were used in accordance with the instructions. To measure plasma GLP-1 levels of the patients participating in the ascending and descending colon biopsy cohort, a Milliplex^®^ Human Metabolic Hormone Panel V3 was used (Cat. No.: HMH3-34K, Millipore, Madrid, Spain) in accordance with the instructions provided.

### 2.6 Statistical analysis

Our results are expressed as mean ± standard error of the mean (SEM). P-values < 0.05 were considered statistically significant. Shapiro-Wilk test was utilized to determine if the data distribution was parametric or non-parametric. We used Student’s T test when comparing two groups or one-way ANOVA with Bonferroni *post hoc* test when comparing multiple groups, for parametric data. For non-parametric data we worked with Mann–Whitney U test, when comparing two groups, or Kruskal–Wallis test by ranks with Dunn’s Multiple Comparison *post hoc* test when comparing multiple groups. Test used in individual figures is stated in the footnote of the pertaining figure. All calculations were performed using Lumivero XLSTAT 2023.1.5 software (Addinsoft, New York, NY, USA).

## 3 Results and discussion

### 3.1 *TAS2R14*, the most promiscuous bitter taste receptor, was one of the most abundant ones at the main GIT locations

To investigate the sensing of bitter taste receptors for modulating the gut-brain axis, a comprehensive analysis of the distribution of 26 distinct TAS2R receptors along the gastrointestinal tract (GIT) is needed. This will enable a more refined delineation of the receptors that are predominant and therefore more promising targets for activation. In this study we met this challenge by conducting a comparative assessment of *TAS2R* gene expression at various GIT locations. We used three different cohorts of patients, and demographic parameters of each cohort can be found in (**Table 1**). We used quantitative RT-PCR as this tool was shown to be optimal for this task (21). However, even though our approach enables comparison of the bitter receptors at each location, it does not enable comparison of abundances between the segments.

**Table 1:** Demographic information about the individual cohorts that have participated in the analysis of the expression of bitter taste receptors.

	Chek Mucosa	Jejunal Mucosa	Colon Mucosa
N	25 ♂	21 ♀	25 (14 ♂, 11 ♀)
Age	65.2 ± 10.7	46.1 ± 11	63.6 ± 6
Weight (kg)	68.7 ± 18	116.9 ± 5	75.9 ± 14
Height (m)	1.67 ± 0.09	1.64 ± 0.09	1.68 ± 0.1
BMI	24.4 ± 4.9	43 ± 4.7	26.7 ± 3.3

**Figure 1:**
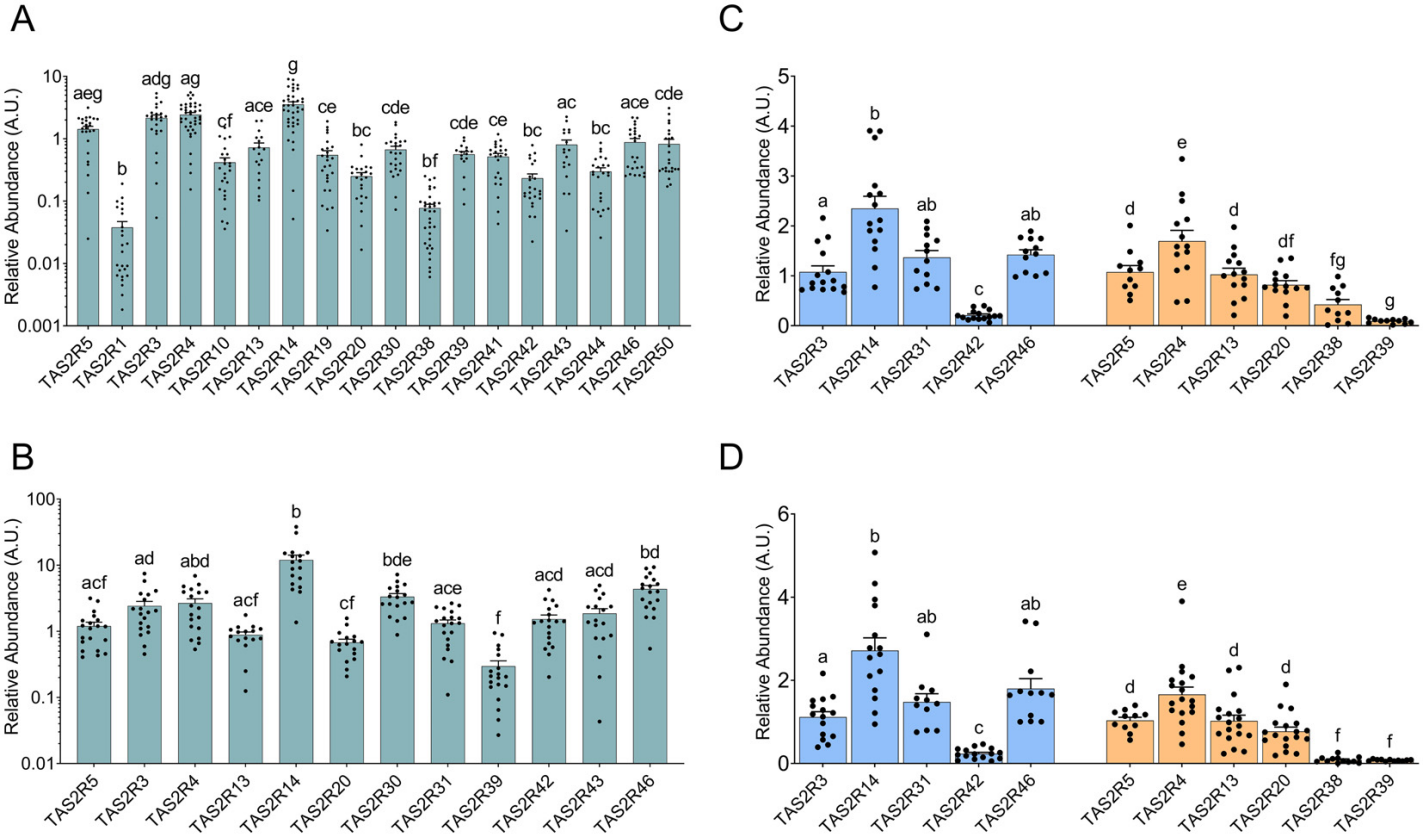
Relative abundances of TAS2R along the GIT vs abundance of the first TAS2R in each graph (either TAS2R5 or TAS2R3) from mouth mucosa **(A)**, jejunum **(B)**, ascending colon mucosa **(C)** and descending colon mucosa **(D)**. **(A, B)** show date in log scale for Y axis. The two colors used in Panels **(C, D)** signify two different measurements and cannot be compared. The groups that share the same letters are not significantly different (significant p value: p<0.05) as determined by the *post hoc* test (we used non-parametric Kruskal–Wallis test by ranks with Dunn’s Multiple Comparison *post hoc* test for **(A, B)**, and parametric one-way ANOVA with Bonferroni *post hoc* test for **(C, D)**; n=11-40).

First, we analyzed TAS2R expression in cheek mucosa. (**Figure 1A**) shows the relative abundances of 18 *TAS2R* in a group of men aged 65 ± 11. *TAS2R14* showed the highest abundance, followed relatively close by *TAS2R3*, *4* and *5*. *TAS2R1* and *TAS2R38* showed the lowest abundances, with the remaining *TAS2R* displaying varying intermediate levels. No other studies have compared the abundances of the various TAS2R in cheek mucosa. Most studies have examined inflammatory processes in pulp tissues where TAS2R stimulation would be useful for anti-inflammatory purposes or dental pulp repair (22), oral microbiome regulation (23), or histamine regulation (24).

Next, we analyzed the jejunum, assessing the relative abundance of the top 12 *TAS2R* as described previously (12) in a group of women with morbid obesity aged 46 ± 11. (**Figure 1B**) displays the *TAS2R* profile from this group, with *TAS2R14* and *TAS2R46* again being the most abundant, and *TAS2R39, 20, 5*, and *13* the least. This profile resembles the one analyzed by Liszt et al. (12), which was obtained from cultured jejunal crypts from patients with obesity. The main difference was higher *TAS2R46* expression in our results compared to *TAS2R43*, while Liszt et al. (12) showed the opposite. The main difference between these two studies is that we worked with frozen whole jejunum while Liszt et al. (12) analyzed *TAS2R* expression in the primary cultures of isolated jejunum crypts from donors with morbid obesity. Another study that compared different *TAS2R* in the intestine is that by Mori et al. (21), who quantified the 25 *TAS2R* isoforms in commercial intestine (without clarifying which intestinal segment was used). Our results share a similar relative expression profile to theirs in ten receptors. The differences were limited to *TA2R20* and *TAS2R39*, whose relative expressions were lower in our study. One of the possibilities why these differences could be the fact that we used tissues from patients from obesity, a factor that can have an influence on the genetical expression (15). However, these observed discrepancies are minimal.

**Figure 2:**
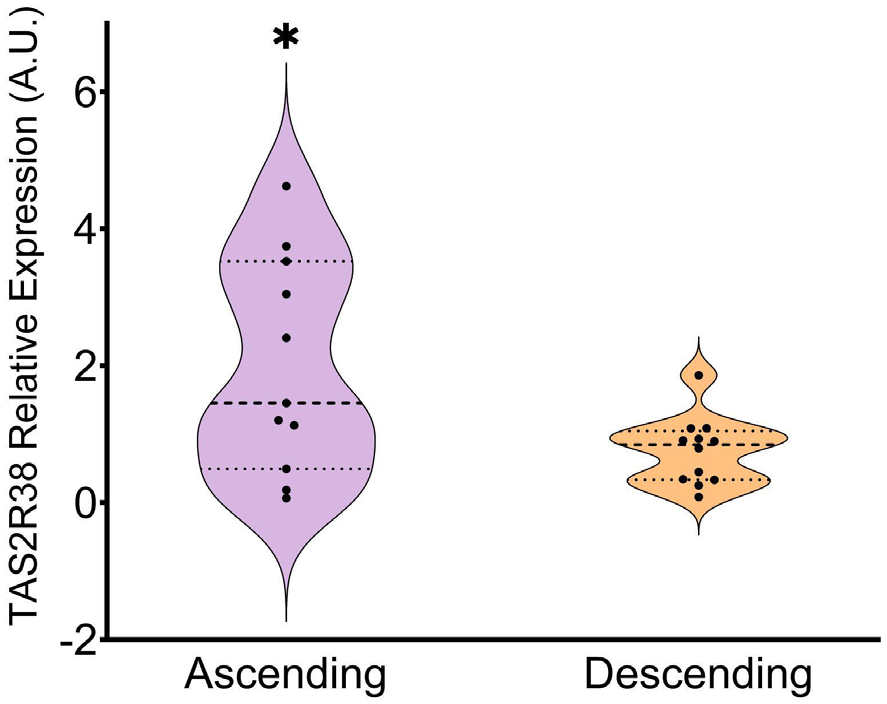
Relative expression of TAS2R38 in the ascending vs descending colon. Patients from the ascending and descending colon mucosa cohort (* indicates p<0.05; parametric Student’s T-test; n=11-12).

Finally, we analyzed colon mucosa from both genders in a third group of subjects aged 63.6 ± 6. As done previously, we analyzed the receptors already described as the most abundant ones in the colon (25). (**Figure 1C**) shows that, in the ascending colon, TAS2R14 again showed the highest presence, along with TAS2R31, 46 and 4, while TAS2R42, 38 and 39 showed the lowest. The only study available on TAS2R expression to compare our results with is that of Rozengurt et al. (25). However, comparison to their work was somewhat challenging, as they only showed the intensity of the bands in their quantification of mRNA, and they do not show TAS2R14 expression. Our results differ to theirs regarding the expression of TAS2R3, 4, 13 and 49, as we obtained lower relative expressions. We obtained a closer result to Rozengurt et al. (25) when we compare the expression levels of TAS2R38, 39 and 42. (**Figure 1D**) shows the profile obtained for the descending colon with the same group of subjects as was used for (**Figure 1C**). Here we can see that relative TAS2R abundance had a similar pattern in both areas of the colon. TAS2R4 and TAS2R14 showed the highest abundance while TAS2R38, TAS2R39 and TAS2R42 showed the lowest levels. Comparison of TASR abundance in the ascending and descending colon showed that only TAS2R38 was statistically less expressed in the descending colon than in the ascending colon (**Figure 2**). Ours is also the first study to compare both colon locations in this regard. Previous studies on this topic are scarce and do not define a specific colon sampling location.

In this context we emphasize the importance of characterizing the precise expression profiles of TAS2R receptors throughout the gastrointestinal tract (GIT) and highlighting the distinctions among these profiles within specific GIT segments. By meticulously mapping the distribution of TAS2R receptors in the GIT and elucidating their roles in regulating local and broader physiological systems, it is feasible to achieve the targeted stimulation of specific GIT regions by using tailored ligands for these receptors and produce well-defined physiological outcomes. Moreover, given the diversity of TAS2R receptors, it is conceivable that targeting different receptors may yield different responses. Preliminary evidence suggests there is a connection between the activation of TAS2R receptors in the oral mucosa and improvements in oral pathologies (23). It is therefore plausible to deduce that similar beneficial effects may manifest in other GIT segments where TAS2R receptors are present.

Another crucial aspect when investigating the activation of TAS2R receptors by dietary components is the dynamic transformation these components undergo during digestion and subsequent metabolism, which include modifications induced by enzymes and interactions with the gut microbiota. These transformations can significantly alter the composition of compounds in the oral cavity compared to those in the colon (26, 27). Notwithstanding the inherent complexities associated with this proposition as briefly elucidated here, (**Table 2**) summarizes the relative abundance of the receptors analyzed at each location. *TAS2R14* appears at all locations as the most expressed bitter taste receptor, and interestingly, it is also the most promiscuous receptor defined to date (8, 28, 29). *TAS2R46*, a receptor that predominates in the jejunum, is also highly promiscuous (28, 29). Only two of the most expressed *TASR* were not characterized as highly promiscuous. One of these is *TAS2R3*, which was highly abundant in the cheek and moderately abundant in jejunum, and reported as the least promiscuous receptor by Margulis et al. (29). The other one is *TAS2R31*, which was highly expressed only in the colon and was also considered to be less promiscuous by Bayer et al. (28). Though incomplete [we did not quantify all 26 TAS2R defined to date (30)], our results suggest there is a general need for a GIT response to any bitter signal, while fine tuning and a specific response may be exerted by receptors that are not so highly expressed.

**Table 2:** Colored summary of the relative abundances of different TAS2R in different parts of the GIT.

Degree of expression	Cheek	Jejunum	Ascending Colon	Descending Colon	Legend:	
Highest	TAS2R4	TAS2R4	TAS2R4	TAS2R4	TAS2R1	TAS2R30
	TAS2R14	TAS2R14	TAS2R14	TAS2R14	TAS2R3	TAS2R31
	TAS2R3	TAS2R46	TAS2R46	TAS2R46	TAS2R4	TAS2R38
	TAS2R5	TAS2R30	TAS2R31	TAS2R31	TAS2R5	TAS2R39
Average	TAS2R10	TAS2R3	TAS2R3	TAS2R3	TAS2R10	TAS2R41
	TAS2R13	TAS2R31	TAS2R13	TAS2R13	TAS2R13	TAS2R42
	TAS2R19	TAS2R42	TAS2R5	TAS2R5	TAS2R14	TAS2R43
	TAS2R43	TAS2R43	TAS2R20	TAS2R20	TAS2R19	TAS2R46
	TAS2R30				TAS2R20	TAS2R50
	TAS2R39					
	TAS2R41					
	TAS2R46					
	TAS2R50					
Lowest	TAS2R38	TAS2R5	TAS2R38	TAS2R38		
	TAS2R1	TAS2R39	TAS2R39	TAS2R39		
	TAS2R42	TAS2R13	TAS2R42	TAS2R42		
	TAS2R20	TAS2R20				

This table is a qualitative summary of **Figure 1**. Each receptor is assigned a different color to facilitate visualization of the differences between locations.

### 3.2 *TAS2R* expression was modified by ageing in the descending colon, while gender differences appeared only in the ascending colon

It is well documented that ageing modifies taste perception, leading to a loss of taste (31, 32). However, most studies have not related this loss of taste to *TAS2R* expression. To investigate whether these effects are also reflected in extraoral TAS2R, we compared *TAS2R* expression in the colon samples of two groups from the same cohort used in previous experiment containing both ascending and descending colon samples: the above-mentioned group of older patients (63.6 ± 6) and a group of younger patients (38.9 ± 6).

**Figure 3:**
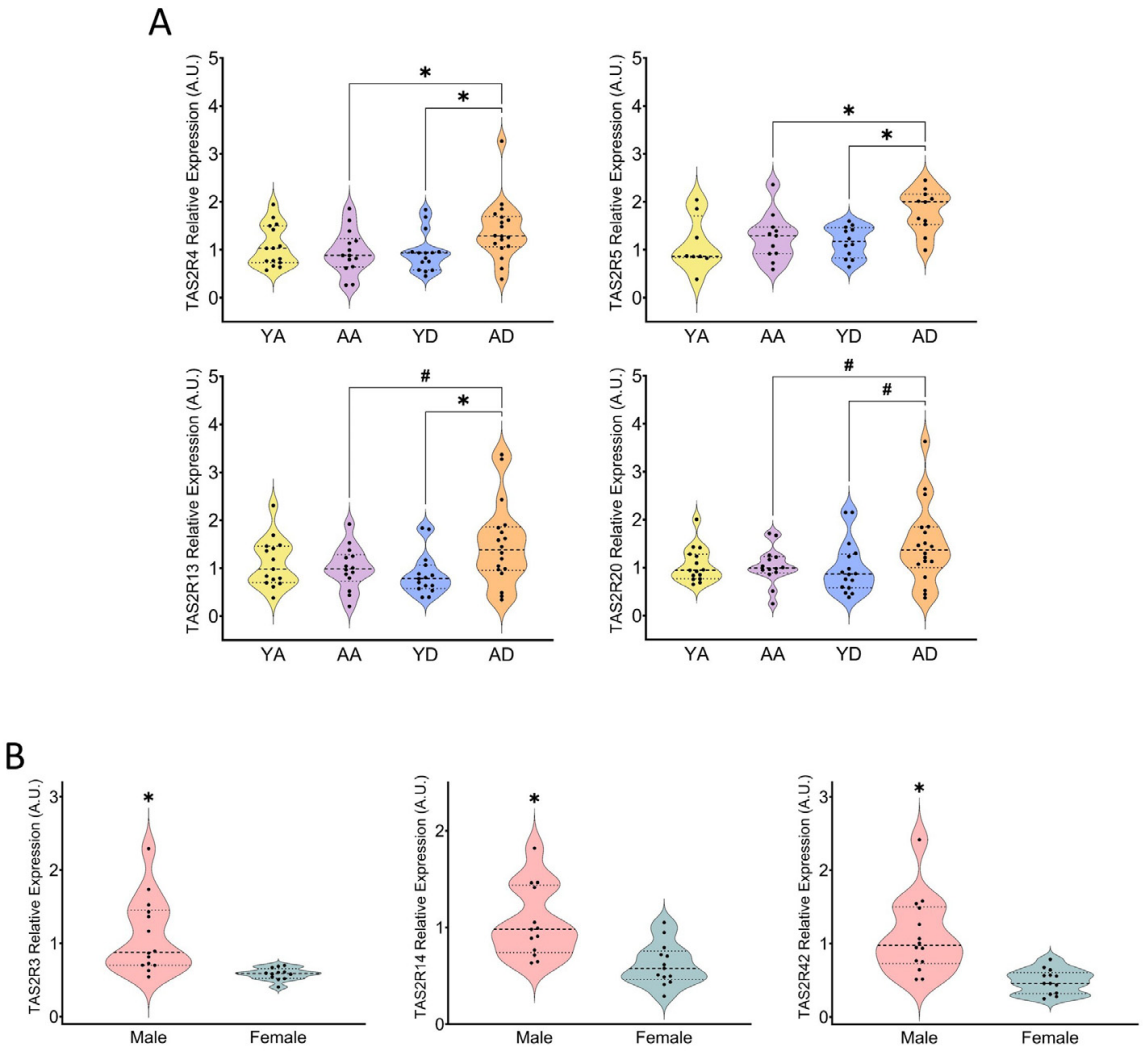
Comparison of relative expression of individual TAS2R in the young and aged groups of the ascending and descending colon **(A)** (YA, young ascending; AA, aged ascending; YD, young descending; AD, aged descending). Gender comparison of relative expressions of specific TAS2R in the ascending colon in all patients **(B)**. Patients from the ascending and descending colon mucosa cohort (* indicates p<0.05; ns indicates 0.05<p<0.1; all the data represented in **(A)** is non-parametric; in 3B, TAS2R3 and TAS2R14 data was parametric, while TAS2R42 was non-parametric; tests used: Student’s T-test, Mann–Whitney U test; n=8-18).

Reduced taste perception may indicate decreased sensitivity to taste-producing molecules. Our findings indicate that ageing does not universally lead to a down-regulation of *TAS2R* expression. In the ascending colon, age groups showed no differences in the abundances of the *TAS2R* analyzed (**Supplementary Table S2**). In the descending colon, there were differences between the analyzed receptors only in the cases of *TAS2R4*, *TAS2R13* and *TAS2R5*, which showed higher expression levels in the older group than in the younger one. The same trend was observed in the expression of *TAS2R20* (**Figure 3A**). Since most of these receptors are not the promiscuous ones, we mentioned earlier (28, 29), ageing might not modify the general response to bitter agonists, but it could impact specific functions of these receptors in the colon. Studies on TAS2R and ageing are limited. One study found a positive correlation between ageing and *TAS2R* expression in non-smokers aged 20 to 65 (33), supporting our findings of increased expression of some receptors with ageing. Another study explored *TAS2R* and ageing through genotype variants and longevity (34). However, most research has focused on aspects other than TAS2R expression, such as age-dependent changes in the physiology of taste receptor cells, related to taste perception loss and ageing (31). Our results therefore suggest that ageing may affect specific TAS2R in a manner distinct from down-regulation and may be specific to certain GIT regions.

Since this colon donor cohort included both genders, we were able to analyze the effects of gender on *TAS2R* expression. There was no gender effect observed in the descending colon (**Supplementary Table S3**). (**Figure 3B**) shows that, in the ascending colon (age groups merged together), we observed a higher quantity of *TAS2R3*, *14* and *42* in males than in females, which suggests that males are more sensitive to the stimulation of these receptors. Regrettably, due to data limitations, we cannot compare our results with other studies. Regarding taste perception, it has been shown that females are more sensitive to PTC (Phenylthiocarbamide) bitterness than males, possibly because women had more fungous papillae than taste buds (35). Regarding the *TAS2R* expression, however, the only study we found described gender differences that were observed only in certain areas of the skin, only for certain receptors, and dependent upon exposure to the sun (36).

### 3.3 Relationship between TAS2R and colonic enterohormones

Some extraoral bitter taste receptors have been shown able to control enterohormone secretion (37–39). To explore this functionality at a deeper level, we analyzed the relative presence of *PYY*, *GCG*, *GLP-1R*, *ghrelin* and *ChgA* by quantifying gene expression in the same samples of colonic mucosa from the human ascending and descending colon used previously to measure *TAS2R* expression.

**Figure 4:**
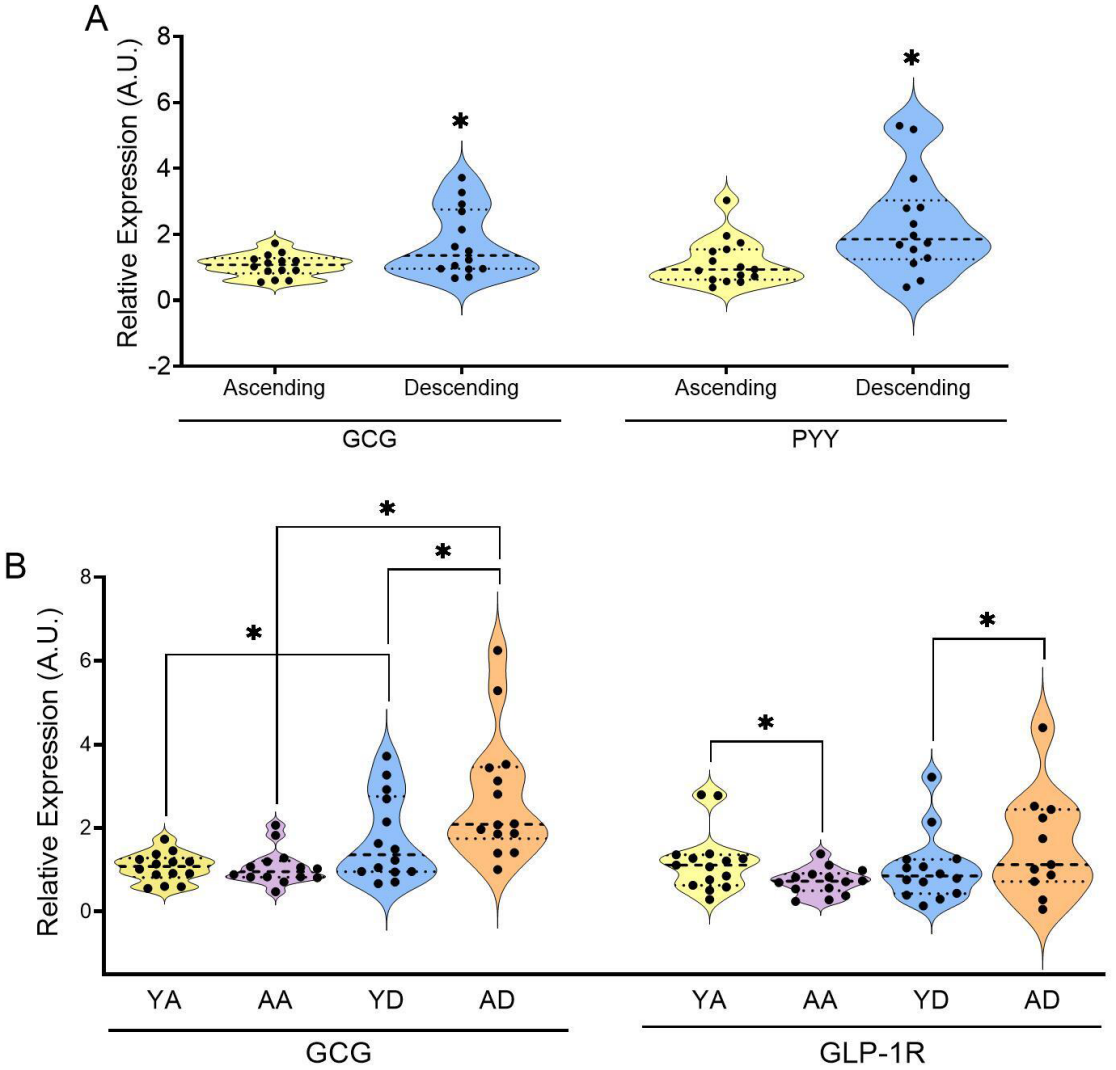
Relative expression of specified enterohormones in the colon samples of the young group of **(A)** and relative expressions of GLP-1 and its receptor in the colon samples of all groups **(B)** (YA, young ascending; AA, aged ascending; YD, young descending). Patients from the ascending and descending colon mucosa cohort (* indicates p<0.05; all the data was non-parametric; test used: Mann–Whitney U test; n=11-15).

In (**Figure 4A**) we can observe location-based differences in enterohormone expression in the younger group. *GCG* was more abundant in the descending colon than in the ascending colon, though the receptor for *GLP-1 (GLP-1R)* showed no significant differences between locations (**Supplementary Figure 1**). A clear location-based effect was also observed for PYY abundance (greater in the descending colon than in the ascending colon). Additionally, *ghrelin* tended to be higher in the ascending colon, while abundance of *ChgA* tended to be higher only in the descending colon when we joined the 2 age cohorts together (**Supplementary Figure 2**). These higher levels of *PYY* expression in the descending colon than in the ascending colon was also observed in human (40), and in mouse and pig (39) tissues in addition to *GCG* displaying the same trend in both humans and mice (39).

To relate expression levels with the secretion of enterohormones, we used a different group of mucosae samples (41) to analyze basal, unstimulated GLP-1 and PYY secretion *ex-vivo* using free explants. In colonic samples we observed that the location significantly influenced PYY secretion, with the descending colon exhibiting higher levels compared to the ascending colon (**Table 3**). However, the same result was not observed for GLP-1 basal secretion. Similar responses were observed for PYY and GLP-1 (**Supplementary Table S4**) in basal, unstimulated secretion measured using an Ussing chamber system.

**Table 3:** Analysis of basal PYY and GLP-1 secretion obtained from colon tissue explants (* signifies statistically significant differences between the two segments of the colon for each enterohormone in each experiment; Student’s T-Test; n= 9).

	Ascending Colon	Descending Colon
PYY (pM)	52 ± 7.23	236.17 ± 65.94*
GLP-1 (pM)	36.92 ± 10.36	41.24 ± 8.1

Since *TAS2R38* was the only TAS2R sensitive to colon location, we conducted a correlation analysis between it and *PYY* and *GCG* expression levels in the descending colon (**Supplementary Table S5**), which yielded no significant effect.

To further explore the relationship between *TAS2R* expression and enterohormones in the colon, we examined how ageing affects enterohormone expression. (**Figure 4B**) shows that *GCG* mRNA was affected by ageing mainly in the descending colon, increasing the differences between the ascending and the descending colon. Ageing led to a statistically significant decrease in *GLP-1R* expression in the ascending colon. However, ageing did not affect the levels of *PYY*, *ghrelin* or *ChgA* (**Supplementary Table S6**). This differential *GCG* gene expression was not strongly associated with the abundance of enteroendocrine cells, as indicated by *ChgA* expression, which showed no age-related differences in both colon locations, therefore indicating no changes in the abundance of cells. These results differ from those previously obtained by our group in a study of 24-month-old female rats (42), where, when working in the proximal colon, we clearly found that ageing decreased *GCG*, *PYY* and *ChgA* expressions. The discrepancy between these results may be related to different causes. Regarding ageing effect, rats were older than the humans analyzed here. As mentioned earlier, ageing increased the sensitivity of the descending colon to stimulation by *TAS2R4, 5, 13* and *20*. Combined with the increased *GCG* abundance and higher basal PYY secretion, this suggests the descending colon is more susceptible to ageing effects. In this study we analyzed the correlation between plasma active GLP-1 (**Supplementary Table S7**) and measured TAS2R. (**Table 4**) shows the correlation analysis conducted in the older group, where we obtained positive correlations with three of the *TAS2R* sensitive to ageing (*TAS2R4, 5, 13*) and *TAS2R39* in the ascending colon. This reinforces the sensitivity of these receptors to ageing and indicates a greater ability to secrete active GLP-1 when the ascending colon is more sensitive to these specific TAS2R stimulations.

**Table 4:** Significant correlation results of the receptors in the ascending colon indicated with plasma levels of GLP-1 (Spearman’s Rho test; * indicates p < 0.05).

	Correlation Coefficient	P value	N
TAS2R4	0.621*	0.024	13
TAS2R5	0.709*	0.022	10
TAS2R13	0.610*	0.027	13
TAS2R39	0.794*	0.006	10

### 3.4 Specific TAS2R stimulation induced GLP-1 and/or PYY secretion

Several studies that specifically stimulated TAS2R reported increased calcium mediator in various cell types (6, 43) but did not report further effects on enterohormone secretion. Working with the samples we used earlier for the basal secretion experiments, we observed a clear connection between TAS2R and enterohormone secretion (41). We stimulated colon tissue samples with an extract rich in procyanidins (GSPE) containing both agonists and antagonists with different specificities for some TAS2R (43, 44). We found that both the ascending and descending colon were sensitive to GSPE stimulation, resulting in increased PYY secretion, but found no effect on GLP-1 secretion.

**Figure 5:**
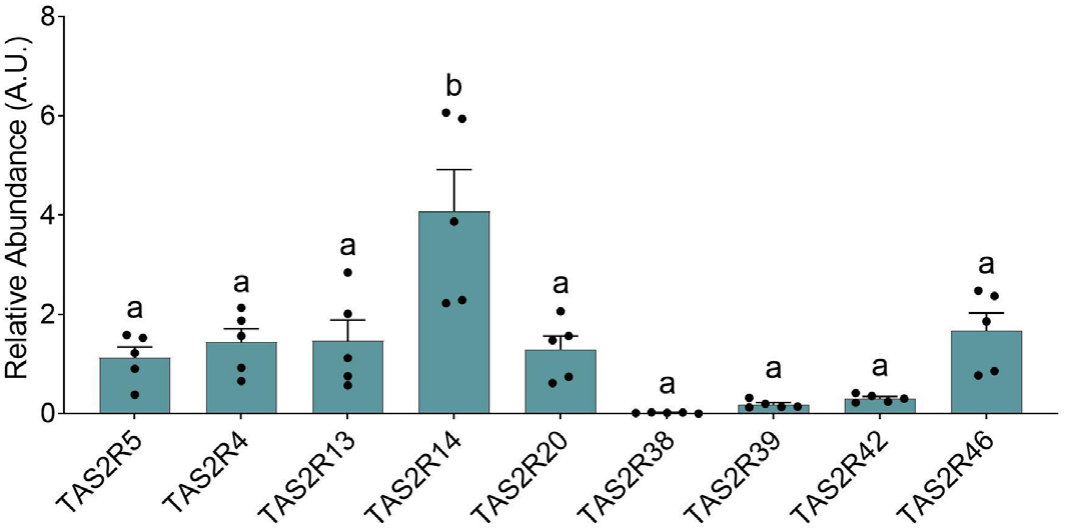
Relative abundances for specific TAS2R obtained from the HuTu-80 cells under basal conditions. The groups that share the same letters are not significantly different (significant p value: p<0.05) as determined by the *post hoc* test (all the data was parametric; one-way ANOVA test with Bonferroni *post-hoc* test; n=5).

To clearly relate TAS2R as integral components of enteroendocrine control, it was necessary to simplify the system. To do so, we selected the HuTu-80 cell line, an enteroendocrine epithelial cell line derived from male duodenal tissue. We decided upon the use of this line as duodenum is location where, yet unmodified compounds arrive to the mucosa and interact with the chemosensory peptides located in the epithelium, and spend significant time, thus enabling the interactions between them. In (**Figure 5**), which shows the relative abundance of the most abundant *TAS2R* in these cells, the receptors analyzed were those previously reported as being the most abundant in these cells, and our results were mostly consistent with those observed by Rozengurt et al. (25). One discrepancy between their results and ours concerns *TAS2R38*, which they observed to be moderately expressed (closer to *TAS2R13*) but which we found barely detectable. Another discrepancy concerned *TAS2R5*, as our data suggest that the expression levels for this receptor were closer to those for *TAS2R4*, *13, 20* or *46*, whereas Rozengurt et al. (25) reported very modest expression closer to the *TAS2R39*. Mori et al. (21) presented a quantitative TAS2R profile for Hutu-80 cells, showing relatively lower abundances of *TAS2R4* and *TAS2R46* and a higher abundance of *TAS2R42* when compared to our data. If we compare the TAS2R profile obtained from our cells with those for the various intestinal segments analyzed in this paper (see **Table 2**), we see that the profile is very similar to that obtained from the jejunum samples. This is as expected for cells of duodenum origin, such as those of the HuTu-80 cell line.

To investigate the stimulation of specific enterohormones in the HuTu-80 cell line, we chose ligands with preferential binding to specific TAS2R. We selected vanillic acid as the specific agonist for TAS2R14 and epicatechin to stimulate TAS2R5 and TAS2R39 (45). Before conducting the experiments, we discarded the toxicity effect of the assayed doses by LDH viability assay (data not shown). (**Table 5**) shows that vanillic acid, at the concentration of its EC50 (44), clearly stimulated GLP-1 secretion in the cells but did not stimulate PYY. Epicatechin 500 μM, which binds preferentially to TAS2R5 (EC50 = 3.2 mM) rather than to TAS2R39 (EC50 = 3.8 mM) (43), showed the ability to induce GLP-1 and PYY secretion in these cells, which, according to Rozengurt et al., expressed higher levels of *GCG* and lower levels of *PYY* (25). Stimulation of the secretion of GLP-1 but not of PYY after activating TAS2R14 is a result we observed in previous studies when, interestingly, we used meat and insect hydrolysates in human colon samples (46). In fact, we obtained the same result in our cells when we used peptone as a positive control (see **Table 5**). Supporting this idea are the results obtained by Kohl et al. (47) in a screening study in which they activated different TAS2R using various peptides and showed that the peptides positively stimulated only receptors TAS2R1, 4, 39, 14 and 46 but did not stimulate TAS2R5. This reinforces the idea that specific TAS2R stimulation produces differential enterohormone secretions. Our results show that TAS2R14 stimulation induces GLP-1 secretion and that TAS2R5 stimulation can induce GLP-1 and PYY secretions.

**Table 5:** Analysis of PYY and GLP-1 secretion obtained after specific TAS2R stimulation in Hutu-80 cells.

	GLP-1 secretion (A.U.)	PYY secretion (A.U.)
Control	1.00 ± 0.08	1.10 ± 0.07
Peptone (5mg of protein/mL)	3.84 ± 0.90*	0.98 ± 0.12
Vanillic Acid 150 μM	1.97 ± 0.33*	1.12 ± 0.94
Epicatechin 500 μM	1.79 ± 0.22*	1.41 ± 0.03*

The data represents the average value for each treatment of each hormone, normalized to the values of the control (* signifies statistically significant stimulation by the treatment vs control group; Student’s T- Test; n= 9).

In summary, our study elucidated variations in *TAS2R* expression profiles at three gastrointestinal (GIT) locations while highlighting their modulation by age and gender, especially in the colonic region. Although samples derived from different types of patients are a potential limitation of the work, this profiling provides valuable insights into the potential dissimilar sensitivities of each location to dietary components and their ensuing metabolites post-GIT transit. Combining this knowledge with a broader understanding of TAS2R-mediated control over enteroendocrine secretion will advance our comprehension of gut-brain communication and inform the development of targeted strategies aimed at modulating these receptors in response to intestinal compounds. Notably, we observed that *TAS2R14*, receptor recognized as the most promiscuous one, exhibited the highest abundance in the four GIT segments analyzed. However, its role in regulating PYY secretion appears to be minimal. Conversely, TAS2R5, a less prevalent but more specific TAS2R, demonstrated the capacity to stimulate both GLP-1 and PYY secretions. Moving forward, the imperative lies in establishing definitive associations between specific TAS2R and cellular functions. This would represent a pivotal milestone in characterizing distinct bitter taste receptors as viable therapeutic and preventive healthcare interventions.

## Data Availability

The data used to obtain the results presented in this article can be found in the repository CORA (https://dataverse.csuc.cat/), under the same title as the article itself (https://dataverse.csuc.cat/dataset.xhtml?persistentId=doi:10.34810/data1647).
