# Supplementary material for: Profiling bitter taste receptors (TAS2R) along the gastrointestinal tract and their influence on enterohormone secretion. Gender- and age-related effects in the colon
Source: Front Endocrinol (Lausanne). 2024 Oct 17;15:1436580. doi: 10.3389/fendo.2024.1436580 (PMC11541047; doi:10.3389/fendo.2024.1436580)
Supplement: Supplementary file 1 [file DataSheet1.docx]

Supplementary Material

Profiling Bitter Taste Receptors (TAS2R) along the Gastrointestinal Tract and their Influence on Enterohormone Secretion. Gender- and Age-Related Effects in the Colon

**Florijan Jalševac; Maria Descamps-Solà; Carme Grau-Bové; Helena Segú; Teresa Auguet; Francesc Xavier Avilés-Jurado; Francesc Balaguer; Rosa Jorba; Raúl Beltrán-Debón; Maria Teresa Blay; Ximena Terra; Montserrat Pinent1*; Anna Ardévol**

**Correspondence:** Corresponding Author: [montserrat.pinent@urv.cat](mailto:montserrat.pinent@urv.cat)

**Appendix A1: Exclusion criteria used in each cohort:**

- Exclusion criteria for the samples of human cheek mucosa, kindly provided by Joan XXIII University Hospital (Tarragona, Spain): (1) serious systemic disease such as obesity, cancer or severe kidney or liver disease; (2) systemic diseases with intrinsic inflammatory activity; (3) history of liver disease (chronic active hepatitis or cirrhosis) and/or abnormal liver function (alanine transaminase and/or aspartate transaminase three times above the upper normal value) or altered renal function (creatinine>1.5mg/dL); (4) adherence to a vegetarian or irregular diet; (5) severe disorders of eating behaviour; (6) clinical symptoms and signs of infection in the previous month; (7) anti-inflammatory chronic treatment with steroidal and/or nonsteroidal anti-inflammatory drugs; (8) antibiotic treatment in the previous three months; (9) major psychiatric antecedents; and (10) uncontrolled alcoholism or drug abuse.
- Exclusion criteria for the jejunum samples, kindly provided by GEMMAIR from IISPV and Joan XXIII University Hospital (Tarragona, Spain): (1) intake of ethanol or other toxins above 10 g/day; (2) acute or chronic hepatic or inflammatory disease, infectious disease or neoplastic disease; (3) menopause or use of contraceptives; (4) diabetes with administration of insulin or other medication to modulate endogenous insulin levels; and (5) administration of fibrates.
- Exclusion criteria for the mucosal biopsy samples from the ascending and the descending colon, kindly provided by the Hospital Clínic (Barcelona, Spain): (1) body mass index (BMI) below 18.5 or above 35; (2) diagnosis of diabetes mellitus type 1 or 2; (3) chronic treatment with anti-inflammatory medicines; and (4) previously diagnosed illnesses.
- Exclusion criteria for the mucosa colon samples, kindly provided by Joan XXIII University Hospital (Tarragona, Spain): (1) alcohol intake above 30 g/day; (2) body mass index above 40 kg/m2; (3) use of drugs unrelated to treatment for metabolic syndrome; (4) presence of intestinal malabsorptive or inﬂammatory bowel disease; (5) presence of acute or chronic inﬂammatory or infectious disease; and (6) presence of neoplastic disease at advanced stages or requiring pharmacological treatment.

**Appendix A2: List of probes tested in the study:**

TAS2R1, Hs00251930_s1; TAS2R3, Hs00249942_s1; TAS2R4, Hs00249946_s1; TAS2R5, Hs01549633_s1; TAS2R10, Hs00256794_s1; TAS2R13, Hs00256781_s1; TAS2R14, Hs00256800_s1; TAS2R19, Hs00853130_s1; TAS2R20, Hs00604340_s1; TAS2R30, Hs03054740_Sh; TAS2R31, Hs00604313_sH; TAS2R38, Hs00604294_s1; TAS2R39 Hs00603443_s1; TAS2R41, Hs00603461_s1; TAS2R42, Hs00704057_s1; TAS2R43, Hs00853105_sH; TAS2R44, Hs00604313_Sh; TAS2R46, Hs00853124_s1; TAS2R50, Hs00604351_s1; GCG, Hs01031536_m1; GLP-1R, Hs00157705_m1; PYY, Hs00373890_g1; CHGA, Hs00900370_m1; Ghrelin, Hs01074053_m1; and RPS9, Hs02339424_g1.

S-table 1: The list of medication that have been prescribed to the participants of the study; Data available for the patients of the colon samples donated by Hospital Clínic from Barcelona. Some patients were prescribed medication for more than 1 medical issue.

| Medical Issues | Number Of Patients Prescribed Medicine |
| --- | --- |
| None | 18 |
| Heart/Circulation-related issues: | 9 |
| Control of stomach acid: | 8 |
| Lowering of Lipids: | 5 |
| Mental Health Problems: | 4 |
| Anticoagulants: | 2 |
| Antihistaminics: | 2 |
| Calcium: | 1 |
| Cancer Prevention: | 1 |
| Contraceptives: | 1 |
| Glaucoma: | 1 |
| Hypothyroidism: | 1 |
| Prostatic Hyperplasia: | 1 |
| Pulmonary Disease: | 1 |
| Smoking Cessation: | 1 |
| Vitamin D: | 1 |

S-table 2: Evaluating the effect of Age on the Relative expression levels of analysed TAS2R in Ascending colon (either Student’s T-test or Mann–Whitney U test was used).

|  | Young | | | Aged | | |
| --- | --- | --- | --- | --- | --- | --- |
| Receptor | Average (A.U.) | SEM | N | Average (A.U.) | SEM | N |
| TAS2R3 | 1.065 | 0.140 | 11 | 1.246 | 0.139 | 15 |
| TAS2R4 | 1.078 | 0.112 | 15 | 0.955 | 0.122 | 14 |
| TAS2R5 | 1.119 | 0.200 | 8 | 1.256 | 0.151 | 11 |
| TAS2R13 | 1.109 | 0.132 | 15 | 1.004 | 0.120 | 14 |
| TAS2R14 | 1.101 | 0.171 | 11 | 1.242 | 0.130 | 15 |
| TAS2R20 | 1.053 | 0.094 | 15 | 1.031 | 0.103 | 14 |
| TAS2R31 | 1.131 | 0.184 | 7 | 0.825 | 0.084 | 12 |
| TAS2R38 | 1.196 | 0.336 | 8 | 1.990 | 0.472 | 11 |
| TAS2R39 | 1.127 | 0.155 | 8 | 1.073 | 0.156 | 11 |
| TAS2R42 | 1.305 | 0.307 | 12 | 1.372 | 0.161 | 15 |
| TAS2R46 | 1.080 | 0.167 | 7 | 1.045 | 0.069 | 12 |

S-table 3: Evaluating the effect of Gender on the Relative expression levels of analysed TAS2R in Descending colon (either Student’s T-test or Mann–Whitney U test was used).

|  | Male | | | Female | | |
| --- | --- | --- | --- | --- | --- | --- |
| Receptor | Average (A.U.) | SEM | N | Average (A.U.) | SEM | N |
| TAS2R3 | 0.734 | 0.075 | 12 | 0.936 | 0.101 | 13 |
| TAS2R4 | 1.512 | 0.205 | 20 | 1.335 | 0.175 | 20 |
| TAS2R5 | 1.145 | 0.088 | 10 | 1.412 | 0.195 | 14 |
| TAS2R13 | 1.482 | 0.213 | 14 | 1.236 | 0.195 | 20 |
| TAS2R14 | 0.843 | 0.073 | 12 | 0.977 | 0.120 | 13 |
| TAS2R20 | 1.543 | 0.217 | 14 | 1.130 | 0.159 | 20 |
| TAS2R31 | 0.832 | 0.069 | 10 | 1.255 | 0.345 | 7 |
| TAS2R38 | 0.522 | 0.115 | 11 | 0.426 | 0.085 | 14 |
| TAS2R39 | 1.039 | 0.107 | 10 | 1.103 | 0.118 | 14 |
| TAS2R42 | 0.801 | 0.082 | 12 | 0.844 | 0.138 | 13 |
| TAS2R46 | 0.938 | 0.080 | 10 | 1.176 | 0.251 | 7 |

**S-figure 1**: The relative expression of the GLP-1 receptor in the ascending and descending colon of the young cohort. No differences were observed (Student’s T-test; n=11-15).


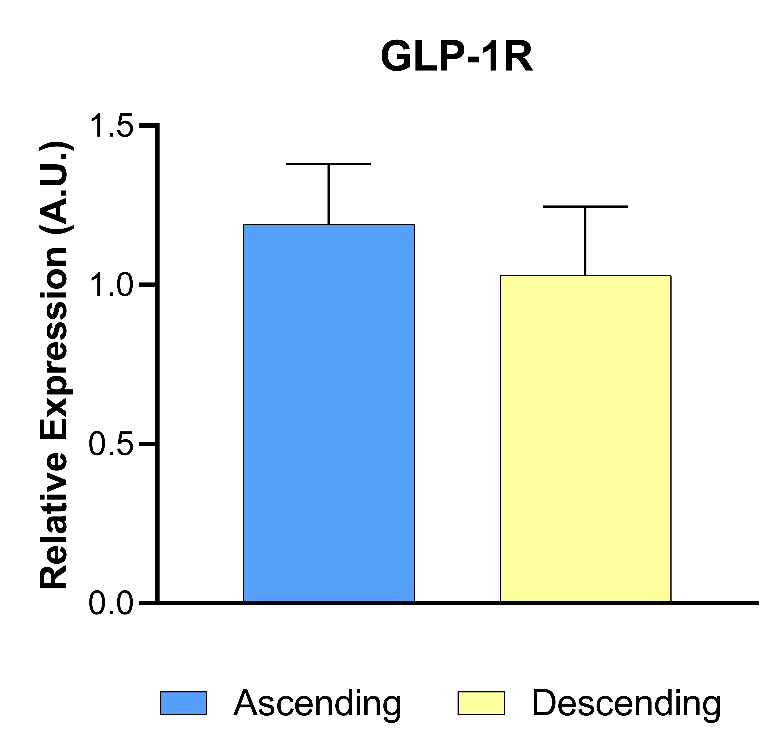


**S-figure 2**: The relative expression of the Ghrelin and ChgA in the ascending and descending colon of the participants; young and aged groups merged together (# indicates 0.05<p<0.1; Mann–Whitney U test; n=29).


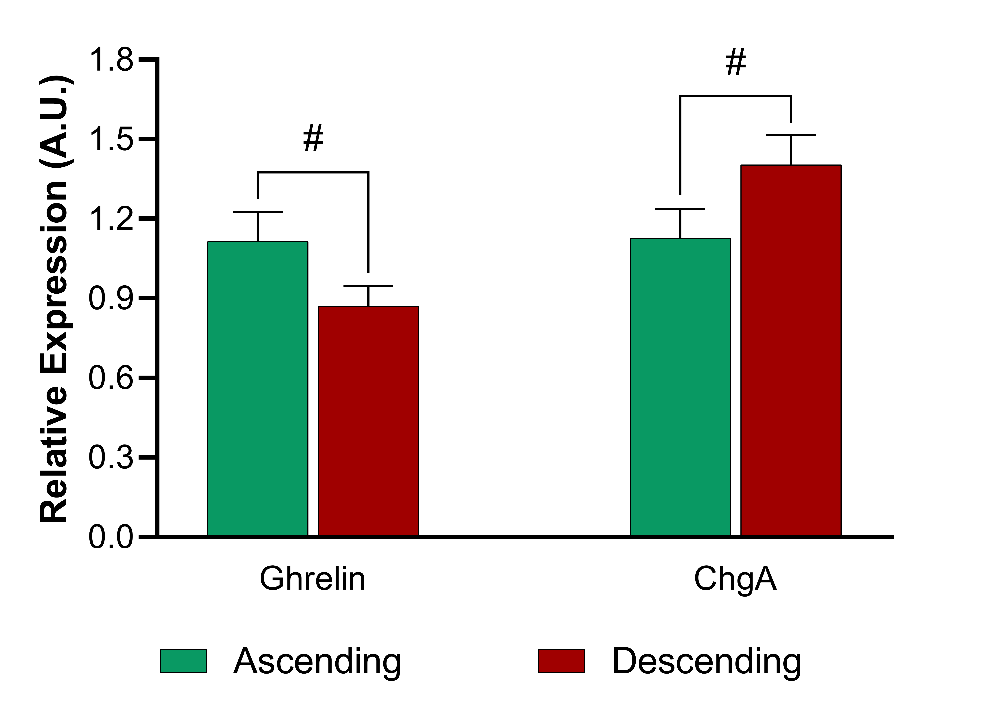


S-table 4: Unstimulated enterohormone secretions from ex vivo intestinal segments of human colon, obtained in the Ussing chamber experiment (* signifies statistically significant differences between the two segments of the colon Student’s T-Test; n= 9-13).

|  | Ascending Colon | Descending Colon |
| --- | --- | --- |
| PYY (pM) | 10.70 ± 3.14 | 35.85 ± 3.4* |
| GLP-1 (pM) | 5.92 ± 2.05 | 5.56 ± 1.67 |

S-table 5: Correlations analysis between expression of TAS2R38 in the descending colon with the expressions of the GCG and PYY in both ascending and descending colon (Spearman's Rho test).

|  | Correlation Coefficient | P value | N |
| --- | --- | --- | --- |
| GCG Expression in Ascending Colon | -0,129 | 0,633 | 16 |
| GCG Expression in Descending Colon | -0,297 | 0,325 | 13 |
| PYY Expression in Ascending Colon | 0,009 | 0,974 | 16 |
| PYY Expression in Descending Colon | -0,423 | 0,150 | 13 |

S-table 6: Evaluation of the effect of Ageing on the Relative expression levels of analysed Enterohormones in Each individual location of the colon (either Student’s T-test or Mann–Whitney U test was used).

|  | | Young | | | Aged | | |
| --- | --- | --- | --- | --- | --- | --- | --- |
| Receptor | Colon Location | Average (A.U.) | SEM | N | Average (A.U.) | SEM | N |
| PYY | Ascending | 1.161 | 0.180 | 15 | 0.795 | 0.072 | 14 |
|  | Descending | 2.315 | 0.406 | 14 | 2.890 | 0.456 | 15 |
| Ghrelin | Ascending | 1.153 | 0.160 | 15 | 0.906 | 0.122 | 14 |
|  | Descending | 0.820 | 0.107 | 13 | 0.798 | 0.094 | 15 |
| CHGA | Ascending | 1.104 | 0.129 | 14 | 0.997 | 0.116 | 14 |
|  | Descending | 1.303 | 0.163 | 14 | 1.261 | 0.138 | 15 |

S-table 7: Evaluation of Plasma levels of GLP-1 in the ascending/descending colon cohort (p<0.05; Mann–Whitney U test).

|  | Young | | | Aged | | |
| --- | --- | --- | --- | --- | --- | --- |
| Enterohormone | Average (pg/mL) | SEM | N | Average (pg/mL) | SEM | N |
| GLP-1 | 10.581 | 1.290 | 21 | **21.63 *** | 3.154 | 23 |
